# Functionalized *Tobacco Mosaic Virus* Coat Protein Monomers and Oligomers as Nanocarriers for Anti-Cancer Peptides

**DOI:** 10.3390/cancers11101609

**Published:** 2019-10-22

**Authors:** Coralie Gamper, Caroline Spenlé, Sonia Boscá, Michael van der Heyden, Mathieu Erhardt, Gertraud Orend, Dominique Bagnard, Manfred Heinlein

**Affiliations:** 1 Institut de Biologie Moléculaire des Plantes (IBMP-CNRS), Université de Strasbourg, 67000 Strasbourg, France; coragamper@gmail.com (C.G.); cspenle@unistra.fr (C.S.); sonia.boscasanjose@gmail.com (S.B.); mathieu.erhardt@ibmp-cnrs.unistra.fr (M.E.); 2 INSERM 1119, BMNST Laboratory, Université de Strasbourg, 67000 Strasbourg, France; heyden@unistra.fr; 3 Labex Medalis, Université de Strasbourg, 67000 Strasbourg, France; 4 Fédération de Médecine Translationnelle de Strasbourg, FMTS, Université de Strasbourg, 67000 Strasbourg, France; 5 INSERM 1109, MN3T, The Microenvironmental Niche in Tumorigenesis and Targeted Therapy, Université de Strasbourg, 67000 Strasbourg, France; 6 INSERM 1109, The Tumor Microenvironment Laboratory, Université de Strasbourg, 67000 Strasbourg, France; gertraud.orend@inserm.fr; 7 University of Strasbourg Institute of Advanced Study (USIAS), 67000 Strasbourg, France

**Keywords:** plant virus, tobacco mosaic virus, nanoparticle, nanocarrier, cancer, angiogenesis, neuropilin-1

## Abstract

Components with self-assembly properties derived from plant viruses provide the opportunity to design biological nanoscaffolds for the ordered display of agents of diverse nature and with complementing functions. With the aim of designing a functionalized nanoscaffold to target cancer, the coat protein (CP) of *Tobacco mosaic virus* (TMV) was tested as nanocarrier for an insoluble, highly hydrophobic peptide that targets the transmembrane domain of the Neuropilin-1 (NRP1) receptor in cancer cells. The resulting construct CPL-K (CP-linker-“Kill”) binds to NRP1 in cancer cells and disrupts NRP1 complex formation with PlexA1 as well as downstream Akt survival signaling. The application of CPL-K also inhibits angiogenesis and cell migration. CP was also fused to a peptide that targets the extracellular domain of NRP1 and this fusion protein (CPL-F, CP-Linker-“Find”) is shown to bind to cultured cancer cells and to inhibit NRP1-dependent angiogenesis as well. CPL-K and CPL-F maintain their anti-angiogenic properties upon co-assembly to oligomers/nanoparticles together with CPL. The observations show that the CP of TMV can be employed to generate a functionalized nanoparticle with biological activity. Remarkably, fusion to CPL allowed us to solubilize the highly insoluble transmembrane NRP1 peptide and to retain its anti-angiogenic effect.

## 1. Introduction

Nanoparticles play an ever-increasing role as carriers for transporting drugs to specific tissues and cells to combat diseases [1,2], such as glioblastoma [3] and breast cancer [4] among others. Carrier-mediated drug delivery systems can offer many advantages over delivery of a physical mixture of multiple drugs. The advantages include (1) prolonged half-life in the circulation provided by the carrier, (2) reduced nonspecific uptake, (3) increased accumulation at the tumor site either through passive enhanced permeation and retention (EPR) effects or through active targeting by incorporation of targeting ligands, (4) endocytotic uptake, thereby bypassing multidrug resistance, and (5) “ratio-metric-dosing”, that is, the ability to tailor the relative ratios of each agent based on its pharmacological disposition. Moreover, a single delivery system carrying multiple drugs in the same platform can lead to controlled and synchronised pharmacokinetics of the drugs, thus resulting in an improved treatment efficacy. Also, a single formulation improves solubility and bioavailability. Although many artificial nanoparticle platforms are under development [5], particular attention is given to nanoparticles derived from plant viruses [6,7,8,9,10,11]. Virus-derived nanoparticles are particularly attractive because they are both biocompatible and biodegradable, and their antigenicity can be attenuated by polymer coating [12]. Viral nanoparticles can be designed and engineered by genetic and chemical protocols. Plant viruses (unlike animal or human viruses) represent a safe platform since they do not cause diseases in humans [13]. Their size is in the nanometer range, thus enhancing permeability of tissues and retention in tumors [14,15,16,17]. They are suitable for both chemical and genetic manipulation, allowing the viral coat to be tailored for specific cell or tissue types, imaging purposes, and as a carrier for therapeutic cargo. Their multivalent nature enables the incorporation of multiple molecules with different functions, thus allowing, for example, the combination of a cell targeting ligand and an imaging agent on the same nanoparticle [10].

The rod-shaped *Tobacco mosaic virus* (TMV) has been studied for more than a century [18,19] and is the most economically and scientifically important plant virus [20]. The virus particle is 300 nm long and 18 nm in diameter and is composed of the 6.7 kb long viral RNA genome encapsidated by a helical arrangement of 2130 identical copies of coat protein (CP). The protein has 158 amino acids and its structure is known [21,22,23,24,25]. The particle readily assembles in vitro [26,27,28] with a short stretch of 432 nts of its RNA (OAS, origin-of-assembly) being sufficient for assembly [29]. Without RNA and at neutral pH, the CP assembles into a ”20S aggregate “, a 18 nm double “disk” (or “nano-ring”) comprising two layers of 17 CP units, which already can be used as nanoscale scaffold. Dependent on the applied pH, ionic strength, and temperature, the protein can also be isolated as “Protein A” (a mixture of CP monomers, trimers, and pentamers) or helical rods of various lengths [30,31]. These assemblies can be generated also with CP recombinantly expressed in *E. coli* [32,33,34,35,36,37]. The CP has several accessible sites for chemical modification at the outer and inner surface. The protein also offers the possibility to insert peptides at the N and C-terminus, as well as in a loop containing amino acid residues 59–66, for display on the surface of intact virions or CP assemblies [38]. This latter property is interesting because peptides and in particular cell-penetrating peptides have clear beneficial effects in the context of cancer disease [39,40,41]. Among the different therapeutic approaches in which peptides are used, a recent strategy involves a “membrane-targeting peptide” (MTP) of 30 amino acids that mimics the transmembrane segment of NRP1 (MTP-NRP1). NRP1 is expressed in several human tumors where its high levels are associated with invasive tumor growth and worsened clinical outcome [42,43]. NRP1 is also highly expressed in tumor-associated blood vessels [44]. Blocking NRP1 signaling reduces tumor angiogenesis and tumor growth [45]. In particular, application of MTP-NRP1 was shown to inhibit NRP1 and associated receptors, thereby blocking downstream signaling and reducing tumor angiogenesis [46,47,48].

MTP-NRP1 contains a double canonical GXXXG amino acid motif (G, glycine; x, any amino acid), known to promote and stabilize interactions between transmembrane protein helices. Any mutation of the glycine residues in the GXXXG motif was shown to interfere with the tumor-suppressing activity of the peptide [46]. As compared to the classical approaches with drugs that target the extra- or intracellular domains of NRP receptors [49,50] or their ligand-binding site, inhibition of NRP1-mediated signaling platforms by disrupting the interaction of NRP1 with itself and with other receptors within the membrane represents a novel concept that has been proven to inhibit tumor angiogenesis [47]. However, hydrophobic transmembrane peptides are highly insoluble in aqueous solutions and, therefore, require the presence of detergents for solubilization. Moreover, due to low solubility, the production and purification of the peptides by chemical synthesis is expensive. In addition, although MTP-NRP1 is active only upon integration into the plasma membrane and only acts on tumors, it shows a large biodistribution profile in the whole body [47]. To improve specific delivery at the tumor site, it is mandatory to couple this peptide to a targeting moiety that promotes its incorporation in the membrane of target cells only. A potential additional targeting moiety that could be combined with MTP-NRP1 is a heptapeptide (ATWLPPR; ATW-NRP1) that was shown to compete with VEGFA165 binding to NRP1 [51]. ATW-NRP1 has already been applied in targeted photodynamic therapy by using it for the specific delivery of the photosensitizer to the tumor site, which improved the efficiency of tumor reduction [52,53].

Here, we show a new method for mass production and purification of the highly hydrophobic MTP-NRP1 by fusing it to the CP of TMV (CPL-K, for CP-linker-“kill”). This method was also used to produce a CP fusion protein displaying three copies of ATW-NPR1 in tandem (CPL-F, for CP-linker-“find”). We demonstrate that the fusion proteins are functional and can be assembled into disks, thus leading to the possibility to create multifunctional plant virus-derived nanoparticles in which the function of the different peptides can be used in a combined “find and kill” strategy, thereby enabling the delivery of biologically active peptides to target NRP1-expressing cells.

## 2. Results

### 2.1. Characterization of Coat Protein (CP) Fusion Proteins Produced in Bacteria

**Figure 1:**
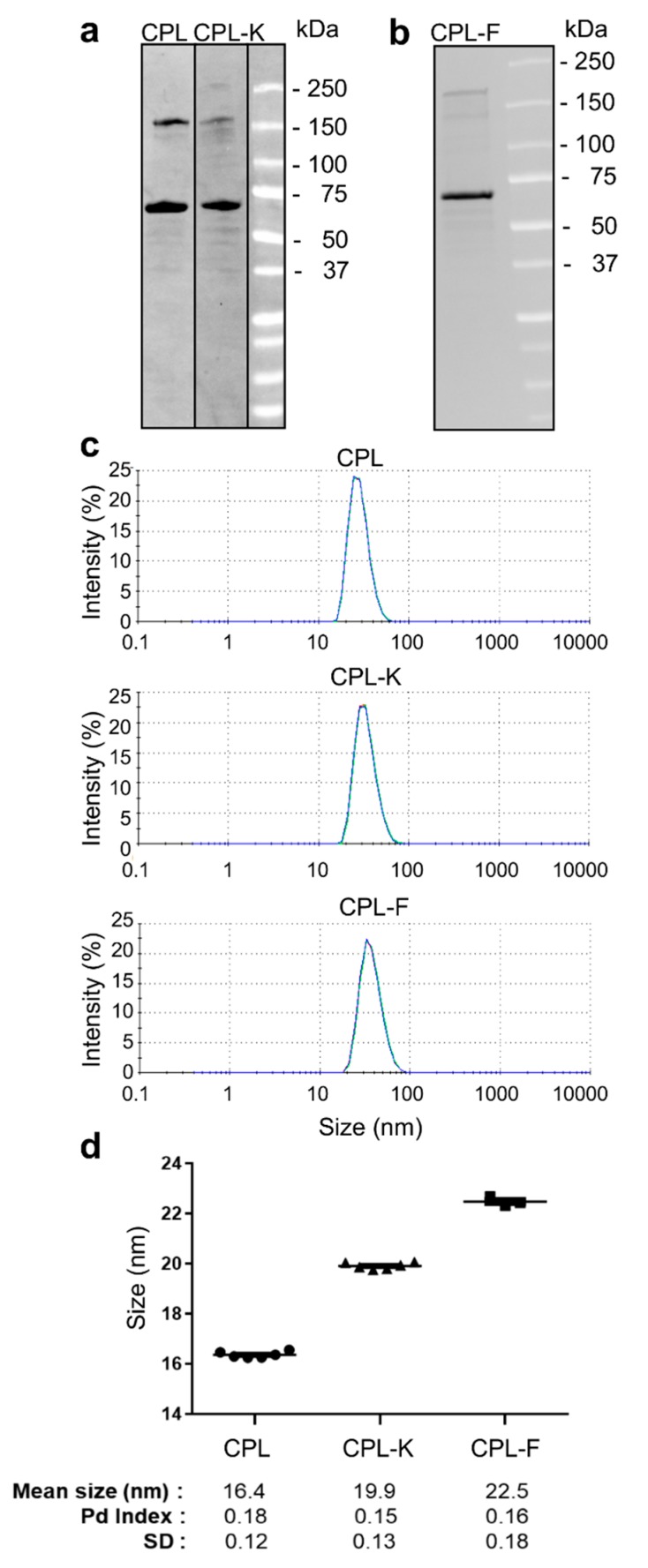
Analysis of CPL, CPL-K and CPL-F. (**a**,**b**) SDS-Page analysis of CPL, CPL-K (**a**), and CPL-F (**b**). The respective molecules are in the range of the estimated sizes of 59 kDa (CPL), 61 kDa (CPL-K) and 62 kDa (CPL-F). (**c**) Dynamic light-scattering (DLS) measurement of CPL, CPL-K and CPL-F. Measurements were performed three times independently for each of six samples per construct. (**d**) Mean particle sizes detected by DLS in the six samples per construct. Pd, polydispersion; SD, standard deviation.

Fusion proteins consisting of CP fused to a linker (L, (GGGGS)_3_) and to the specific peptide at the C-terminus and to a poly-histidine-(His6)-maltose-binding-protein (MBP)-tag at the N-terminus were expressed in *E. coli* and purified on MBP Trap HP columns. As shown in Figure 1a,b, the isolated CP fusion proteins showed the expected molecular weight of 59 kDa for CPL, 61 kDa for CPL-K and 62 kDa for CPL-F, respectively, in stain-free sodium dodecyl sulphate-polyacrylamide gel electrophoresis (SDS-PAGE) gels. Additional bands were also detected around 180 kDa for all constructs, which may presumably be due to multimerization of the proteins. Dynamic light-scattering (DLS) analysis of recombinant proteins (of 6 different preparations) in potassium phosphate buffer PPB (pH 8; 22 °C) revealed that CPL, CPL-K and CPL-F form monodispersed solutions (Figure 1c) of small particles with a hydrodynamic radus of 16.4 ± 0.12 nm (polydispersion (Pd) index = 0.18) for CPL, 19.9 ± 0.13 nm (Pd index = 0.15) for CPL-K, and 22.5 ± 0.18 nm (Pd index = 0.16) for CPL-F (Figure 1d).

### 2.2. CPL-K Interacts with NRP1 and Inhibits NRP1 Binding to Plexin A1

**Figure 2:**
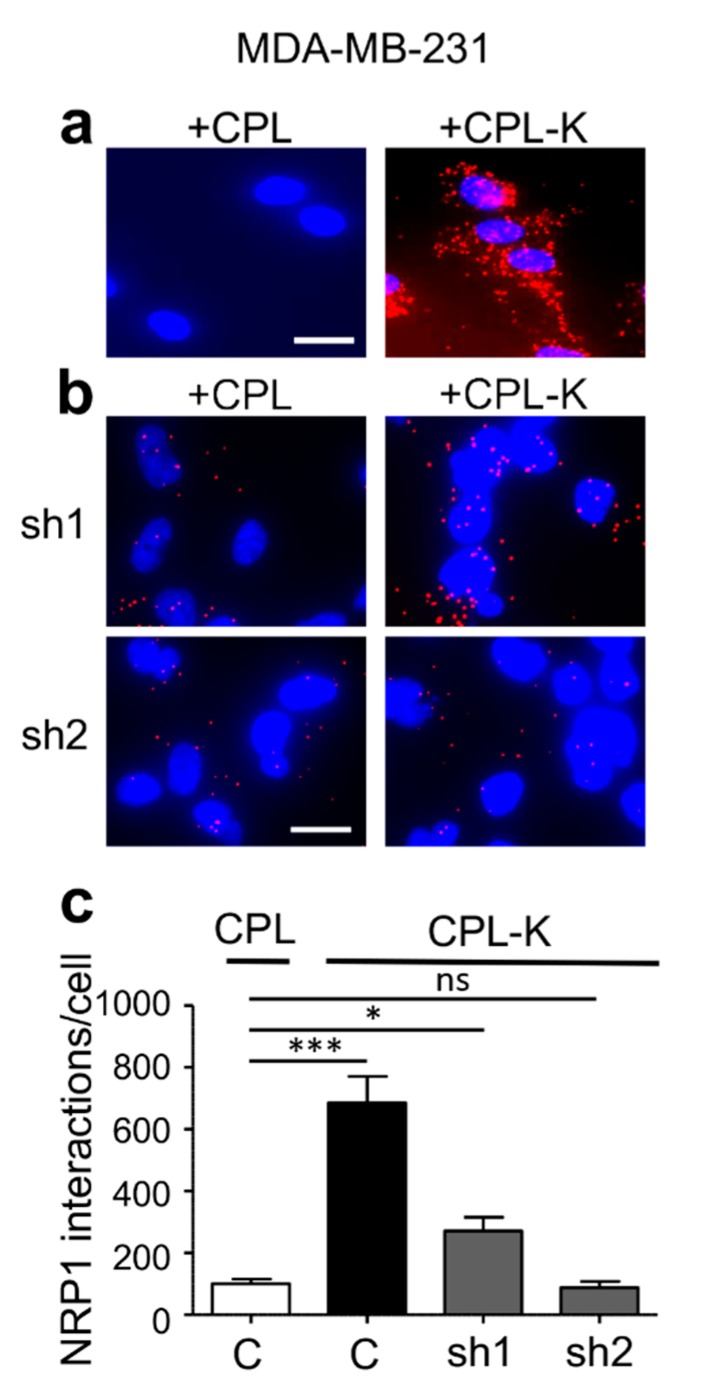
CPL-K interacts with Neuropilin-1 (NRP1) in MDA-MB-231 cells. (**a**,**b**) Proximity ligation assays (PLA) with MDA-MB-231 cells treated either with 1 µM CPL or 1 µM CPL-K and using antibodies against the cellular NRP1 protein together with antibodies against the MBP part of CPL and CPL-K. NRP1 forms complexes with CPL-K (red fluorescent dots) but not with the CPL control protein. NRP1/CPL-K complexes are formed in normal cells (**a**) but not in cells of two different cells lines in which NRP1 expression is knocked down (sh1, sh2) (**b**). Scale bar, 10 µm. (**c**) Quantification of NRP1/CPL or NRP1/CPL-K interactions (fluorescent dots per cell) in cell lines expressing shRNA constructs or not (C = control). *N* = 3 experiments, 5 to 10 imaging fields were quantified per condition and replicate experiment. * *p* < 0.05; *** *p* < 0.0005, ns > 0.05 (non-parametric analysis of variance (ANOVA) test followed by Dunn’s multiple comparison test).

**Figure 3:**
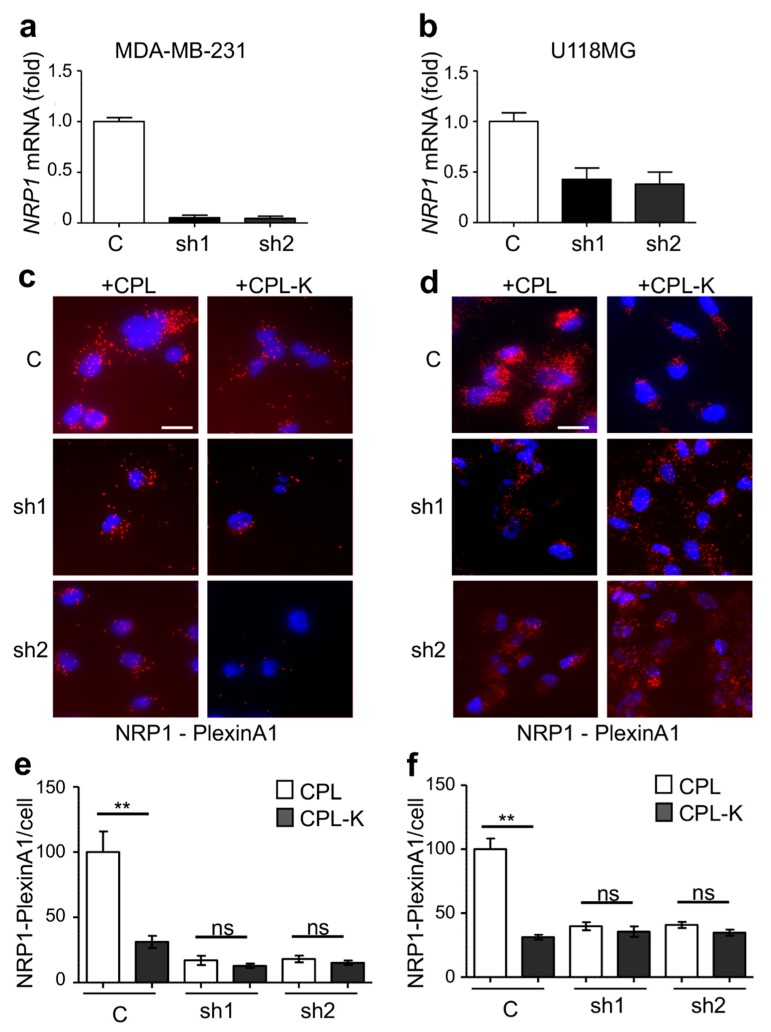
CPL-K disrupts NRP1/PlexinA1 complex formation. (**a**,**b**) Downregulation of NRP1 with shRNA constructs (sh1, sh2) in MDA-MB231 cells (**a**) and U-118MG cells (**b**) as determined by RT-qPCR. (**c**–**f**) Imaging (**c**,**d**) and quantification (**e**,**f**) of NRP1/PlexinA1 complex formation as determined by PLA with antibodies for NRP1 and PlexA1 in the presence of 1 µM CPL or CPL-K in MDA-MB-231 (**c**,**e**) and U-118MG cells (**d**,**f**) and, upon knockdown of NRP1. Scale bar, 20 μm. ** *p* < 0.005; ns = not significant (one-way ANOVA and Dunn’s multiple comparison test).

We investigated whether CPL-K binds to NRP1 in living cells with a fluorescent proximity ligation assay (PLA) applied to cultured MDA-MB231 cells that naturally express NRP1. We used specific antibodies binding to MBP and NRP1, respectively, to reveal the interaction between the recombinant protein and the target receptor. According to the atomic model of CP [22], the N- and C-termini of the CP are very close together. Thus, the N-terminally fused MBP is an excellent reporter for the interaction of the C-terminally fused MTP-NRP1 with NRP1-containing receptor complexes in the membrane of target cells. As shown in Figure 2a, only a few fluorescent spots corresponding to non-specific binding were detected when 1 µM CPL was added to the cells. However, numerous spots were counted when MDA-MB231 cells were treated with 1 µM recombinant CPL-K protein, thus reflecting the high capacity of CPL-K to bind NRP1. The specificity of the interaction was assessed in two independent shRNA-expressing MDA-MB231 cell lines in which *NRP1* was silenced. Indeed, both cell lines (sh1 and sh2) showed a significantly lower number of spots, suggesting that the amplification signal (red dot) is generated when CPL-K interacts with NRP1 (Figure 2b,c). To further demonstrate the interaction of CPL-K with NRP1, we investigated whether CPL-K can inhibit the dimerization of NRP1 with PlexinA1. Therefore, we determined the number of NRP1/Plexin-A1 dimers at the surface of the wild-type (WT) cells and in cells knocked down for *NRP1*. This assay was performed with cells of the glioblastoma U-118MG cell line previously shown to express NRP1 and Plexin-A1 [54], cells of the metastatic breast cancer cell line MDA-MB231, and cells with a knockdown of *NRP1* expression that was confirmed by reverse transcription followed by quantitative polymerase chain reaction (RT-qPCR) analysis in both cell lines (Figure 3a,b). Using the PLA with NRP1 and PlexinA1 antibodies, numerous fluorescent spots were obtained when MDA-MB231 and U-118MG cells were incubated with CPL, thus demonstrating that the high level of NRP1/PlexinA1 dimers is not altered by CPL. Incubation of the cells with CPL-K, however, significantly reduced the number of spots, thereby demonstrating the disruption of NRP1/Plexin-A1 dimers in both cell types. As expected, cells of the respective *NRP1* knockdown lines showed only a low number of spots also in the presence of CPL, confirming the specific detection of NRP1/Plexin-A1 dimers and their disruption by CPL-K. Quantification of the spots revealed that CPL-K disrupted the NRP1/Plexin-A1 dimers in the WT cells to the level observed in the *NRP1*-silenced cells. Taken together, these observations indicate the capacity of CPL-K to bind NRP1 and to interfere with the dimerization function of NRP1.

### 2.3. CPL-K Inhibits VEGFA-Induced Tumor Cell Migration and Human Umbilical Vein Endothelial Cell (HUVEC) Tubulogenesis

**Figure 4:**
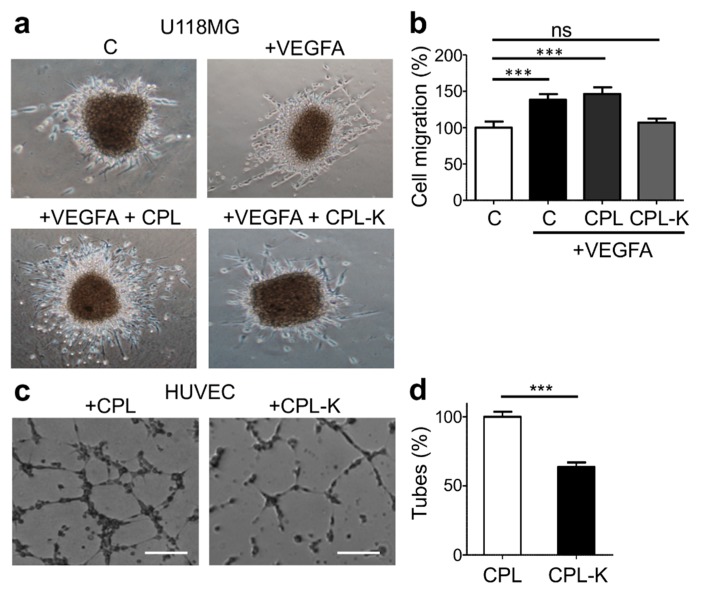
CPL-K inhibits cell migration and angiogenesis. (**a**,**b**) Phenotype (**a**) and quantification (**b**) of U-118MG cell migration in a 3D coagulated chicken plasma matrix after 24 h of culturing the cell aggregate. Results are normalized to the cell migration observed in medium without VEGFA. The relative surface of migration (%) was determined with Image J. N = 3 independent experiments, 5–30 explants measured per condition for each experiment. *** *p* < 0.0001 ** *p* < 0.001 Mann-Whitney test. (**c**,**d**) Phenotype (**c**) and quantification (**d**) of human umbilical vein endothelial cell (HUVEC) tubulogenesis on Matrigel 4 h after seeding in complete medium. Scale bar, 200 μm. Results are normalized to tubulogenesis in the presence of CPL. Note a 37% decrease of tube formation with CPL-K. *N* = 9 independent experiments. *** *p* ≤ 0.0001; ns = not significant (Mann–Whitney test).

We previously showed that the disruption of the NRP1/Plexin-A1 dimer suppresses vascular endothelial growth factor type A (VEGFA)-induced migration of glioblastoma U-118MG cells [54]. To determine whether the CPL-K-induced disruption of NRP1/Plexin-A1 dimers produces similar effects on cell migration, we performed a 3D migration assay. Therefore, U-118MG cell aggregates were grown in plasma clots in the presence or absence of VEGF and in the presence of CPL or CPL-K. As is shown in Figure 4a,b, VEGFA increased cell migration in comparison to medium alone, as demonstrated by the more pronounced total surface occupied by migrating cells around the border of the aggregates after 24 h. Notably, in the presence of CPL-K, VEGFA-induced migration was reduced to levels as seen without VEGFA, which was not the case in the presence of CPL. The disruption of the NRP1/Plexin-A1 complex was previously also shown to inhibit human umbilical vein endothelial cell (HUVEC) tube formation on Matrigel [54]. Interestingly, HUVEC Matrigel tubulogenesis assays showed that CPL-K, but not CPL, has the same effect (Figure 4c,d). Altogether, these results demonstrate that CPL-K inhibits VEGFA-induced migration and HUVEC tubulogenesis. Importantly, the reduction of HUVEC tubulogenesis by 37% indicates that the anti-angiogenic activity of CPL-K is similar to that of free MTP-NRP1, which was shown to inhibit tubulogenesis by 30 % [47].

### 2.4. CPL-F Binds to NRP1 and Inhibits HUVEC Tubulogenesis

**Figure 5:**
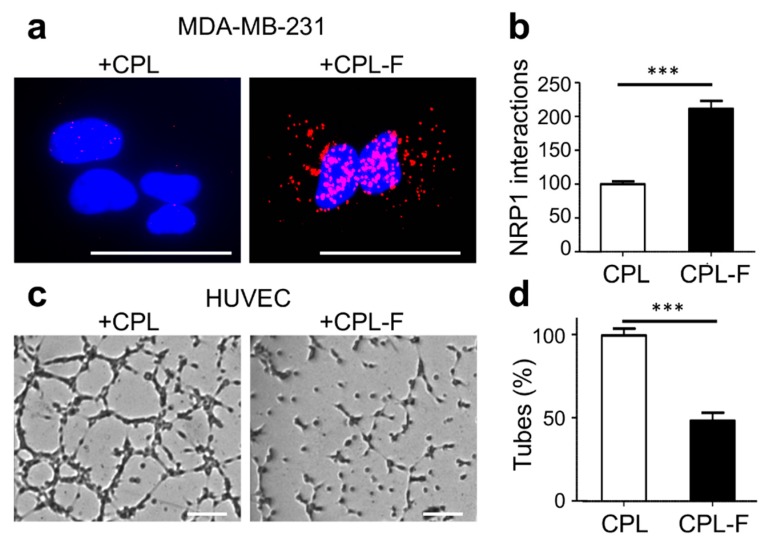
CPL-F interacts with NRP1. (**a**,**b**) Phenotype (**a**) and quantification (**b**) of interaction of CPL and CPL-F in MDA MB231 cells as determined by PLA with antibodies against NRP1 and MBP. Scale bar, 10 µm. Quantification of NRP1/CPL and NRP1/CPL-K complexes per cell. *N* = 3 independent experiments with 5–10 imaging fields analyzed per condition. *** *p* < 0.0001 Mann-Whitney test. (**c**,**d**) Phenotype (**c**) and quantification (**d**) of tube formation of HUVECs four hours after treatment with CPL or CPL-F. Scale bar, 200 μm. Note a 52% decrease in the formation of tubes with CPL-F. *N* = 4 experiments. *** *p* < 0.0001 (Mann–Whitney test).

The heptapeptide ATWLPPR was shown to bind NRP1 and to facilitate cellular uptake of a coupled photosensitizing agent [52,53]. To test the ability of this peptide to guide a tagged CP towards NRP1, we produced recombinant CPL-F (Find), which carries three consecutive modules of the ATWLPPR peptide fused to the C-terminus of CP-L. By PLA using antibodies for MBP and NRP1, we addressed whether CPL-F finds NRP1 and indeed revealed a significant number of interactions between CPL-F and NRP1 (Figure 5a,b). Next, we wanted to know whether CPL-F had an effect on HUVEC tubulogenesis, which was again addressed in a Matrigel tubulogenesis assay. As is shown in Figure 5c,d, CPL-F caused a 52% reduction of HUVEC tube formation.

### 2.5. CPL-K and CPL-F Inhibit NRP1-Dependent Sema3A-Induced Downstream Signaling

**Figure 6:**
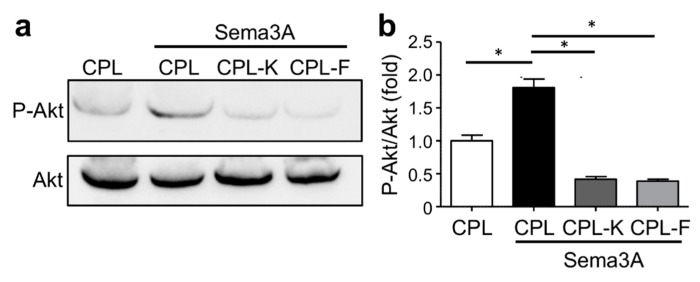
CPL-K and CPL-F inhibit Sema3A-induced Akt phosphorylation. (**a**,**b**) Representative Western blot (**a**) and quantification of Western blot signals (**b**) of Akt and P-Akt in MDA-MB-231 cells upon stimulation with Sema3A. Note that Sema3A-induced P-Akt levels are suppressed by CPL-K and CPL-F but not by CPL. *N* = 3 experiments. * *p* < 0.05 (one-way ANOVA and Dunn multiple comparison test).

As Sema3A binds and activates NRP1, we investigated whether CPL-F and CPL-K affected the phosphorylation of Akt (P-Akt), which is an indicator of downstream signaling [55]. By detection of P-Akt with specific antibodies in Sema3A-stimulated MDA-MB-231 cells, we found that Sema3A stimulated the phosphorylation of Akt in the presence of CPL but not in the presence of CPL-K and the CPL-F (Figure 6). Thus, unlike CPL, CPL-K and CPL-F inhibit the ability of Sema3A to stimulate the Nrp1 signal transduction pathway leading to Akt phosphorylation.

### 2.6. A Nanoparticle Formulation of CPL/CPL-K/CPL-F Inhibits HUVEC Tubulogenesis

**Figure 7:**
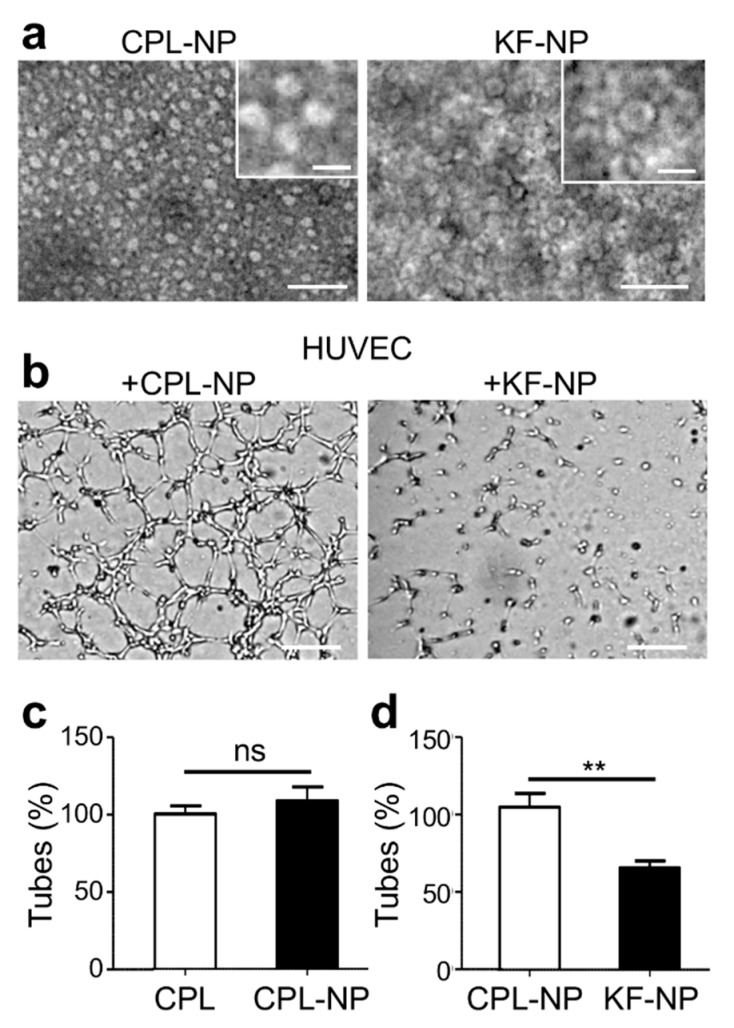
Characterization of monotype and mixed CPL nanoparticles by transmission electron microscopy (TEM) and effect on angiogenesis. (**a**) TEM images of nanoparticle assemblies (disks) derived from CPL and from a mixture of CPL, CPL-K, and CPL-F (KF). Scale bar, 100 nm; scale bar in insert figures, 25 nm; (**c**,**d**) Phenotype (**b**) and quantification (**c**,**d**) of the tubule formation by HUVECs in matrigel. The number of tubes per field was measured four hours after cell plating and upon addition of the different CPL assembly formulations. *N* = 3 experiments, five wells quantified per condition for each experiment. Scale bar, 200 μm. Note that CPL and CPL-NP do not inhibit tube formation. In contrast, particles made of the mixture of CPL, CPL-K and CPL-F (KF-NP) significantly reduce tube formation. *N* = 3 experiments. ns > 0.01; ** *p* < 0.001 (Mann–Whitney test).

So far, we have shown that CPL-F and CPL-K are both active in inhibiting endothelial tubulogenesis. However, the two proteins likely act differently since CPL-F binds to the extracellular domain of NRP1, whereas CPL-K interacts with the transmembrane sequences of NRP1. Although both proteins are active on their own, the question arises whether their effects could be potentiated if combined in the same nanoparticle. As described in previous reports [32,33,34,35,36,37], bacterially expressed and purified CP can be assembled into different aggregates, which is dependent on pH, ionic strength, and temperature. In designing the nanoparticle, we reasoned that the active molecules (finding and killing moieties) should be spatially arranged in a way that they could reach their biological target and do not interfere with each other. Therefore, we combined CPL, CPL-K and CPL-F in equimolar ratios each comprising one third of the mix. In adaptation to reported conditions for the assembly of bacterially produced and modified CP [32], we generated disk-like nanoparticles (NPs) by dialyzing CPL as well as the equimolar mixture of CPL, CPL-K, and CPL-F against 100 mM potassium phosphate pH 8.5 and then, in a second step, against the same buffer at pH 6.0 and at a temperature of 4 °C. The presence of disk-like NPs formed from CPL alone (CPL-NPs) or from mixtures of CPL, CPL-K, and CPL-F (KF-NPs) was verified by electron microscopy (Figure 7a). To address whether the KF-NPs retained biological activity, we tested their effect in HUVEC Matrigel tube formation assays. As depicted in Figure 7b–d, the KF-NPs exhibited a significant anti-angiogenic effect, whereas CPL-NPs had no effect.

## 3. Discussion

In this study we addressed the possibility to generate nanoparticles with anti-cancer properties. The general strategy was to merge three different tools providing a targeting mechanism (to find tumor cells), a tumor growth inhibitory mechanism (to kill tumor cells) and a protein scaffold to assemble the two find and kill moieties. To achieve such a find and kill approach we selected two types of peptides that previously have been demonstrated to have targeting (ATW-NRP1] [53]) and inhibitory functions (MTP-NRP1) [47,48] towards NRP1, a key molecule in promoting cancer growth. Both peptides act at the extracellular and intra-membrane levels without requiring cellular uptake and are, therefore, useful for the chosen targeting approach. NRP1 is a multivalent transmembrane receptor interacting with several other transmembrane molecules (mostly receptors) such as Plexin A1 and VEGFR, and soluble binding partners such as Sema3A, amongst others. NRP1 exhibits multiple functions such as promoting cell migration and angiogenesis, two properties that justify targeting NRP1 for tumor-inhibition [44,56]. As NRP1 is overexpressed in several cancer types [42,43,57], it appears as an attractive therapeutic target both for reaching the tumor bed and for blocking tumor-cell expansion in a variety of cancers. Whereas the ATW-NRP1 peptide binds to the ectodomain of NRP1 and has been previously used as a cancer-targeting tool to enhance the photodynamic destruction of brain tumors [52,53,58], the hydrophobic MTP-NRP1 targets the transmembrane domain of the receptor [45] and was shown to efficiently reduce tumor growth in breast- or brain-tumor models [47,48]. However, because of its hydrophobic nature, the production and solubilization of MTP-NRP1 is difficult, thereby, slowing down its development and also presenting a problem in administration. Hence, to succeed in the production of nanoparticles bearing both the ATW and MTP peptides, we had to select a scaffold compatible with the opposite biochemical/biophysical properties of the two peptides. We selected the TMV-derived CP protein that had been linked to a short linker sequence (CP-L) and fused to MBP to enhance solubility for nanoparticle production. This choice was based on previous reports having demonstrated that TMV and TMV-derived proteins can be used as carriers to target and deliver various peptides and proteins [38,59,60]. This approach turned out to be suitable because the CP fusion proteins displaying either the find (ATW) or the kill (MTP) peptide sequences could easily be produced. The production reached a high yield with a concentration in the range of mg protein/mL. In solution, the proteins formed mono-dispersed, individual particles. Importantly, while expected for CP fusion protein carrying the hydrophilic ATW-NRP1 peptide (CPL-F), the MTP-NRP1 displaying protein (CPL-K) was also shown to be completely soluble without any requirement of detergent or solvent that usually is being mandatory for the solubilization of native MTP-NPR1. The removal of the MBP severely impaired the production of CPL-K. This is in line with our first trials using engineered TMV for production of the peptides in plants. Here, the high hydrophobicity of MTP-NRP1 led to sequestration of CP by membranes. This blocked the synthesis of the virions, precluding our attempts to produce CPL-K as part of intact virions in infected plants. Hence, the bacterial approach we established here solves an important issue for the production and solubilization of MTP-NRP1 and potentially other peptides that target membrane domain sequences.

Next, we determined whether the particles conserved the biological properties of the MTP and ATW peptide sequences after fusion with CPL and MBP. The use of a proximity ligation assay (PLA) confirmed that both the CPL-F and CPL-K particles conserved the capacity of the MTP and ATW peptides to bind NRP1. The presence of the MBP tag allowed us to detect the CPL-K at the membrane with specific antibodies, thereby providing the most direct evidence for the interaction of MTP-NRP1 with its target inside the membrane, which previously, with free MTP-NRP1, has been only indirectly possible by examining the disruption of NRP1 complexes by PLA [53]. The specificity of the CPL-F and CPL-K binding to the receptor was confirmed by using cells in which *NRP1* expression was knocked-down. Remarkably, we observed a background signal with CPL suggesting that this protein may stick to the membrane in a non-specific manner. When addressing the interaction of NRP1 with Plexin-A1 at the cell surface, we demonstrated in cultured U-118MG and MDA-MB-231 cells that the CPL-K peptide was able to disrupt this interaction. The disruption of this complex presumably has an impact on the respective downstream signaling. Indeed, by using Sema3A as another NRP1 interactor [61,62], we demonstrated that both CPL-K and CPL-F blocked Sema3A-induced downstream Akt phosphorylation.

As a potential anti-cancer tool, the CPL-carrying peptides should be able to inhibit cancer relevant events. Indeed, we showed that the CPL-F and CPL-K reduced tumor cell migration and angiogenesis, in particular endothelial tube formation. Altogether, these results prove that the CP formulation of the find and kill peptides conserved their biological properties. Next we examined whether we could use the self-assembly property of the CP protein to generate multifunctional particles. In adaptation to the protocol used by Bruckman [32], we produced nano-ring-like structures that we could image by electron microscopy. In terms of solubility and accessibility of the active sites of the find and kill peptides in a mixed nanoparticle, the relative ratio of CPL to the two other peptides might be crucial. We used a one third ratio of each component to minimize cis-interactions between adjacent kill peptides that may inactivate them. This approach represents a good compromise to retain solubility and activity. Nanoparticles created by assembling an equimolar mix of CPL-L, CPL-F, and CPL-K apparently retained a significant anti-angiogenic effect in the HUVEC-based tubulogenesis assays. This amplitude of inhibition was similar to the inhibition already measured for the individual CPL-F or CPL-K monomers. A similar range in inhibition (30–50%) has also been observed after siRNA-based NRP1 silencing [63], thus suggesting that a total inhibition of NRP1-mediated HUVEC tubulogenesis is already reached with single NPs. The chosen approach of mixing the proteins does not guarantee a homogenous and organized distribution of the find and kill sequences within the assembled particles. Future studies that are beyond the scope of this article have to be carried out to modify the ratio and array of CPL-F and CPL-K in mixed nanoparticles. Further studies may lead to potential approaches by which the ratio and array of CPL-F and CPL-K can be determined, optimized, and demonstrated. It can also be expected that the large MBP moieties present on each of the assembled nanoparticle subunits hinders the proper display and optimal accessibility of the adjacent active sequences on the particle surface, which may explain the lack of potentiation that we have observed. Indeed, both the N- and C-terminus of CP are exposed to the TMV particle surface. Thus, while the MBP moiety attached to the N-terminus of CPL may be free in its movements and allow the peptides at the C-terminus access their targets as long as subunits are in monomeric form, particle assembly may impose structural constraints by which MBP is locked in a position by which it may partially interfere with the activity of the peptide at the other end. It will be important to further study the assembly and activity profiles of CPL-K and CPL-F upon removal of MBP or upon application of other solubilizing purification tags. Nevertheless, we demonstrate here an important starting point to produce complex nanoparticles decorated with different peptides by simply mixing the different monomers under conditions favorable for assembly. This concept can now be further developed towards optimization and also for including other peptides with find and kill properties.

## 4. Materials and Methods

### 4.1. Cell Lines

U-118MG and MDA-MB-231 cells were grown in Dulbecco modified Eagle medium (DMEM, Gibco, Thermo Fisher Scientific, Illkirch-Graffenstaden, France) supplemented with 10% fetal calf serum (Gibco, Thermo Fisher Scientific), 100 µg/mL streptomycin, and 100 IU/mL penicillin (Sigma-Aldrich, Saint-Quentin-Fallavier, France). MDA-MB231 RNAi cell lines with reduced *NRP1* expression were generated by using *NRP1*-targeting shRNAs encoded by MISSION Lentiviral transduction particles (SHCLNV-NM_003873, Sigma-Aldrich). As control for the infected cells, a lentivirus carrying a GFP reporter was used (MISSION^®^ TurboGFP™ Control Transduction particles SHC003V, Sigma-Aldrich). Infected cells were selected with puromycin (1 µg/mL). Two of the five different lentiviruses that were used for *NRP1* silencing showed significant reduced expression of *NRP1* (sh1and sh2). *NRP1* silencing was determined by RT-qPCR. Here, total RNA was extracted using TRIzol Reagent^®^ (Invitrogen, Illkirch-Graffenstaden, France) and converted to cDNA with the High-Capacity cDNA Reverse Transcription Kit (Applied Biosystems, Illkirch-Graffenstaden, France. Quantitative PCR was performed using the 7500 Real-Time PCR System (Applied Biosystems) and applying PowerUp™ SYBR™ Green Master Mix together with NRP1 primers gatcATCCTGATCACCATCATCGCTATGTCTGCTCTGGTTGTTCTGCTGGTTGCTGTTTGCGTTGTTGTTCTGTACCGTAAACGT and aattACGTTTACGGTACAGAACAACAACGCAAACAGCAACCAGCAGAACAACCAGAGCAGACATAGCGATGATGGTGATCAGGAT) and the TaqMan™ Fast Advanced Master Mix together with hGAPDH Taqman probe (Hs02786624_g1) for normalization.

### 4.2. Plasmids

Bacterial expression plasmids pHis-MBP-CPL, pHis-MBP-CP-L-sNRP1, and pHis-MBP-CPL-3xF encoding CPL, CPL-K, and CPL-F were created by Gateway^TM^ cloning using sequences of parental constructs pTMV-L, pTMV-L-NRP1, and pTMV-F. pTMV-L-NRP1 was created by replacing a PacI/KpnI fragment of the TMV cDNA (in plasmid pUC3/12; [64]) encompassing nucleotides of CP and the TMV 3’UTR with a synthesized PacI/KpnI fragment (pUC-CP-L-NRP1 based on pUC cloning vector pIDTSMART:AMP; Integrated DNA Technologies, Leuven, Belgium) and containing the same part of TMV but in which DNA encoding a flexible linker peptide (GGGGSGGGGSGGGGS) fused to the DNA encoding the MTP-NRP1 peptide (ILITIIAMSALGVLLGAVCGVVLYRKR) was inserted before the CP stop codon. TMV-L-sNrp1 encoding a shorter version of MTP-NRP1 (GVLLGAVCGVVLYRKR), TMV-L not encoding a targeting peptide, and TMV-F encoding one copy of the ATW peptide (ATWLPPR), were created by PCR using pUC-CP-L-Nrp1 as template. For TMV-L-sNRP1, pUC-CP-L-NRP1 was used together with overlapping primers 5′-AGGCGGTAGTGGCGGAGGGGGTTCCGGAGTTCTCCTTGGTGCCGTCTGTGG-3′ (forward) and 5′-CCACAGACGGCACCAAGGAGAACTCCGGAACCCCCTCCGCCACTACCGCCT-3′ (reverse) to shorten the MTP-NRP1-encoding sequence (sequences encoding part of the NRP1 peptide are underlined). After PCR, the methylated (parental) DNA was removed by digestion with DpnI. To remove the NRP1 peptide-encoding sequence from pUC-CP-L-NRP1 and create pUC-CP-L and pUC-CP-F, the forward primer 5′P-GGTAGTCAAGATGCATAATAAATAACGGATT-3′ was used together with 5′P-GGAACCCCCTCCGCCACTACCGCCTCC-3′ (reverse) or 5′P-TCTAGGAGGAAGCCAAGTTGCAGTTGCAGGACCAGAGGTCCAAACC-3′ (reverse, sequence encoding the ATW peptide ATWLPPR is underlined; “P” stands for phosphorylated), respectively. These primers border the NRP1 sequences to be deleted (for pUC-CP-L) or to be replaced (for pUC-CP-F) on both sides, thus allowing the rest of the plasmid to be amplified. Both primers were phosphorylated at the 5’ end to re-circularize the plasmid by ligation (T4 ligase). The plasmids pUC-CP-L-sNRP1, pUC-CP-L, and pUC-CP-F were digested with PacI and KpnI and the fragments ligated to the digested TMV, creating the constructs TMV-L-sNRP1, TMV-L, and TMV-F. 

To create pHis-MBP-CPL, pHis-MBP-CPL-sNRP1, and pHis-MBP-CPL-3xF, the CPL and CPL-K fragments were amplified from TMV-L-sNRP1 with the primers (attB1 and attB2 recombination sites in bold; TEV protease recognition site in italics) 5’GGGGACAAGTTTGTACAAAAAAGCAGGCTTC*GAAAACCTGTACTTCCAGGGT*ATGGCTTACAGTATCACTACT-3′ (forward) and either 5′-GGGGACCACTTTGTACAAGAAAGCTGGGTTTTAGGAACCCCCTCCGCCACTACC-3′ (for CPL) or 5′-GGGGACCACTTTGTACAAGAAAGCTGGGTTTTACCTCTTTCTATACAATACCACGCC-3′ (for CPL-K) as reverse primer, and cloned into the donor vector pDONR/Zeo (Thermo Fisher Scientific, Waltham, MA, USA) to create pDONR-CP-L and pDONR-CP-L-K. CPL-1F was amplified from TMV-F with primers (attB1 and attB2 recombination sites in bold; TEV protease recognition site in italics) 5′GGGGACAAGTTTGTACAAAAAAGCAGGCTTC*GAAAACCTGTACTTCCAGGGT*ATGGCTTACAGTATCACTACT-3′ (forward) and 5′GGGGACCACTTTGTACAAGAAAGCTGGGTTTTATCTAGGAGGAAGCCAAGTTGC-3′ (reverse, sequence encoding the ATW peptide ATWLPPR is underlined) and introduced into pDONR/Zeo to create pDONR-CP-F. To triplicate the ATW peptide, pDONR-CP-F was re-amplified with primers (ATW-encoding sequence underlined; “P” indicates phosphorylated primer) 5′P-GCAACTTGGCTTCCTCCTAGAGCAACTTGGCTTCCTCC-3′ (forward) and 5′P-TCTAGGAGGAAGCCAAGTTGCAGTTGCAGGACCAGAGGTCC-3′ (reverse), thus adding one additional copy of the ATW sequence to each side of the existing ATW sequence. Following ligation, the resulting plasmid pDONR-CP-3xF was re-amplified with primers (linker sequence is underlined; “P” indicates phosphorylated primer) 5′P-AGGCGGTAGTGGCGGAGGGGGTTCCGCAACTTGGCTTCCTCCTAGA-3′ (forward,) and 5′P-CCACCAGACCCTCCACCTCCAGTTGCAGGACCAGAGGTCC-3′ (reverse) to insert the linker (GGGGSGGGGSGGGGS) in front of the triplicated ATW peptide and creating pDONR-CP-L-3F. The donor plasmids pDONR-CP-L, pDONR-CP-L-K, and pDONR-CP-L-3xF were finally used for recombination with the destination vector pDEST-His-MBP (Addgene, Inc., Cambridge, MA, USA; [65]) to create pHis-MBP-CPL, pHis-MBP-CP-L-sNRP1, and pHis-MBP-CPL-3xF.

### 4.3. Protein Expression and Purification

The recombinant N-terminally His_6_-MBP-tagged CPL proteins were expressed in BL21 (DE3) pLysS (Novagen, Watertown, MA 02472 USA) *E. coli* cells upon selection with 100 µg mL^−1^ ampicillin. Cultures (50 mL or 300 mL) were grown for 40 h at 25 °C in ZYM5052 auto-inducing media. Upon bacterial lysis the proteins were purified on a MBP Trap High Performance (HP) column (GE Healthcare Life Science, Freiburg, Germany) in an ÄKTA Pure chromatography system (GE Healthcare Life Science, Freiburg, Germany) and eluted with 10 mM maltose in phosphate-buffered saline (PBS). Peak fractions were pooled and dialyzed against PBS pH 7.4 using a HiTrap Desalting (DST) column (GE Healthcare Life Science, Freiburg, Germany). Protein concentrations were determined with NanoDrop 2000 UV-Vis equipment (Thermo Scientific, Wilmington, DE, USA). Protein expression and purification steps were monitored by analysis of total, soluble and eluted fractions by SDS-PAGE using pre-casted polyacrylamide gels (PROTEAN TGX Stain-free protein gels, Biorad, Marnes-la-Coquette, France).

### 4.4. Western Blot for Detection of Akt Phosphorylation

Before protein extraction, cells were treated with CPL-K, CPL-F, or CPL at 10^−6^ M for 1 h and then stimulated with Sema3A at 100 ng/mL for 30 min as previously described elsewhere [44]. After lysis in Laemmli buffer (Sigma) supplemented with protease inhibitors (Roche, Basel, Switzerland) and phosphatase inhibitor (sodium orthovanadate, Sigma-Aldrich), protein samples were separated in 4–20% pre-casted polyacrylamide gels (PROTEAN TGX Stain-free protein gels, Biorad) by SDS gel electrophoresis in Tris/Glycine/Sodium Dodecyl Sulfate buffer (Biorad) at 300 V for 18 min. Proteins were transferred onto nitrocellulose membrane using the Trans-Blot^®^ Turbo^TM^ Transfer System (Trans-blot Turbo, Biorad) and antibodies Akt and phospho-Akt (Cell signaling), and their respective secondary antibodies coupled with HRP (Biorad) were used. The blots were developed with ECL (Biorad), imaged with a bio-imager (Chemidoc^TM^ Touch Imaging System, Biorad), and normalized using the stain-free technology (western blot figures can be found in the Supplementary Figure S1).

### 4.5. Dynamic Light-Scattering (DLS) Analysis

The size distribution profile of particles in the protein solutions was measured by DLS using Zetasizer Nano Range ZS equipment (Malvern Panalytical, Malvern, UK). 70 µL of the protein solution (1 mg/mL) was loaded into a cuvette for measurement. The measurements were performed at 22 °C in 0.1M potassium phosphate buffer pH8. Particle sizes were analyzed and then expressed in volume according to the Malvern software instructions.

### 4.6. Proximity Ligation Assay (PLA)

Cells were seeded on PERMANOX slides (Lab-Tec) overnight, and then treated with 10 µM CPL, CPL-K or CPL-F monomers for 1 h. After fixation with 1% para-formaldehyde (PFA) for 10 min, cells were permeabilized with PBS containing 0.1% Triton-x-100. The samples were treated overnight at 4 °C in PBS with appropriate combinations of primary antibodies (mouse anti-NRP1 (Evitria) and rabbit anti-MBP (New England Biolabs, E8031S) for detection of CPL fusion protein binding to NRP1; mouse anti-NRP1 (Evitria) and rabbit anti-PlexA1 (Abcam, ab23391) for detection of receptor protein dimer disruption). Subsequent steps of the assay were performed according to the manufacturer’s recommendations described in the Duolink In Situ Fluorescence Protocol with components of the Duolink PLA and Duolink In Situ Detection Orange kits (Sigma-Aldrich). Finally, cells were mounted with a coverslip using Duolink^®^ In Situ Mounting Medium with (4′,6-Diamidine-2′-phenylindole-dihydrochloride (DAPI, Sigma-Aldrich). Pictures of the labeled cells were taken with an AxioZoom (Zeiss, Axio Imager Z1, Marly le Roi, France) equipped with appropriate light wavelength filters. Fluorescent signals (dots) were quantified with ImageJ software (version 1.52, NIH Bethesda, Maryland, USA).

### 4.7. Cell Migration Assay

Cell migration was analyzed by imaging the number of cells moving away from cell aggregates formed by the hanging drop method [66]. According to this method, U-118MG cells were cultured in UMED medium at 37 °C and under 5% CO_2_ using a T75 flask. Upon reaching 70% confluency, the cells were detached with trypsin (0.05% Trypsin-EDTA 1x, Gibco), collected by centrifugation (5 min; 800 rpm) at room temperature, and resuspended in 150 µL of UMED medium. A Petri dish of 6 cm diameter was filled with 3 mL of culture medium and 20 µL drops of the cell suspension were deposited on the internal part of the lid. The lid was then placed to close the dish and to incubate the cells above the medium at 37 °C and 5% CO_2_ overnight. The following day, aggregates formed by the U188 cells were removed and cut into pieces of 30–50 µm (‘explants’). Next, a 12 × 24 mm glass cover-slip was placed into a 6 cm Petri dish and coated with 20 µL chicken plasma. Subsequently, 15 to 20 ‘explants’ were added onto the plasma. The plasma was then coagulated by addition of 20 µL thrombin followed by incubation at room temperature. Upon completion of coagulation, DMEM medium was added into the Petri dish to cover the cells. CPL or CPL-K was added into the culture medium to a concentration of 10 µM for incubation with the cell aggregates at 37 °C and 5% CO_2_ for 24 h. Microphotographs of the cell aggregates were taken using a Nikon Eclipse TS100 microscope (Nikon, Champigny sur Marne, France) and the area around the aggregates covered with migrated cells was determined with ImageJ software.

### 4.8. Angiogenesis Assay

Human umbilical vein endothelial cells (HUVECs) were cultured at 37 °C under 5% CO_2_ in endothelial cell culture medium (PromoCell, Heidelberg, Germany) supplemented with endothelial cell growth supplement (ECGS; 4 µL/mL), fetal calf serum (FCS, 20 µL/mL, Thermo Fisher Scientific), human epidermal growth factor (hEGF; 0.1 ng/mL), and human basic fibroblast growth factor (hbFGF; 1 ng/mL, Thermo Fisher Scientific). For the assay, plates (15 u-slide Angiogenesis, Ibidi plates, Biovalley, Nanterre, France) were coated with Matrigel (Merck-Millipore, Billerica, MA, USA) at 37 °C for 1 h. Subsequently, 5000 HUVECs in culture medium (50 µL) with the CPL proteins at 10^−6^ M were added to each well for 3 h (37 °C, 5% CO_2_). The cells in each well were imaged by DIC microscopy (Leitz DM RB, Leica, Nanterre, France) and the number of closed tubes was counted for 5 wells per condition.

### 4.9. Disk Assembly

To assemble disk oligomers from hybrid CP monomers, the monomer concentration was set to 2 mg/mL prior to dialysis, as recommended [32]. The samples were initially dialyzed against 100 mM potassium phosphate pH 8.5 at 4 °C for 24 h in a Slide-A-Lyzer MINI dialysis unit (Thermo Fisher Scientific) with a 10 kDa molecular weight cutoff (MWCO). For assembly, the samples were subsequently dialyzed against 100 mM potassium phosphate pH 6 at 4° C for 24 h. To assemble disks from different monomers, equal volumes of the different CPL proteins in 100 mM potassium phosphate pH 8.5 were mixed and the mixed sample was dialyzed against 100 mM potassium phosphate pH 6 for 24 h. Before angiogenesis assays, non-assembled monomers were removed by filtering through Vivaspin 500 columns with a MWCO of 100 kDa (GE Healthcare, Strasbourg, France).

### 4.10. Transmission Electron Microscopy (TEM) Imaging

An 8 µL protein sample was deposited onto a Formvar coated nickel grid for 1 minute. Excess solution was removed with filter paper. The grid was stained with uranyl acetate (15 µL at 2%) and the excess of stain was removed and dried. Subsequently, the sample was observed with a Hitachi H7500 transmission electron microscope (TEM) at 80kV.

### 4.11. Statistical Analysis

Data were analyzed with GraphPad (Prism 5, San Diego, CA, USA). Statistical analyses were performed using Mann-Whitney test (for sample *n* < 30) and one-way analysis of variance (ANOVA) for comparison between groups. Results are given as mean ±SD and considered significant for *p* < 0.05.

## 5. Conclusions

Our data demonstrate that the TMV CP protein fused with MBP protein at its N-terminus and with a flexible linker at its C-terminus can be used as a nanocarrier and scaffold to assemble particles displaying peptides with hydrophilic or hydrophobic properties. This polyvalent platform offers unprecedented possibilities to generate smart nanoparticles in high yields and concentration. Presented here in the form of anti-cancer particles with a find and kill potential, the variation of the types of peptides that can be incorporated is probably unlimited.

## Supplementary Materials

The following are available online at https://www.mdpi.com/2072-6694/11/10/1609/s1, Supplementary Figure S1: Raw data of blot.
