# Supplementary material for: Functionalized Tobacco Mosaic Virus Coat Protein Monomers and Oligomers as Nanocarriers for Anti-Cancer Peptides
Source: Cancers (Basel). 2019 Oct 22;11(10):1609. doi: 10.3390/cancers11101609 (PMC6826726; doi:10.3390/cancers11101609)
Supplement: Supplementary file 1 [file cancers-11-01609-s001.pdf]

Manuscript *Cancers-598226* by Gamper et al.

Raw data of blots shown in Figure 1 and Figure 6

A. Figure 1

Figure 1a and Figure 1b represent to different blots (therefore two figures).

Source data for Fig.S1a:

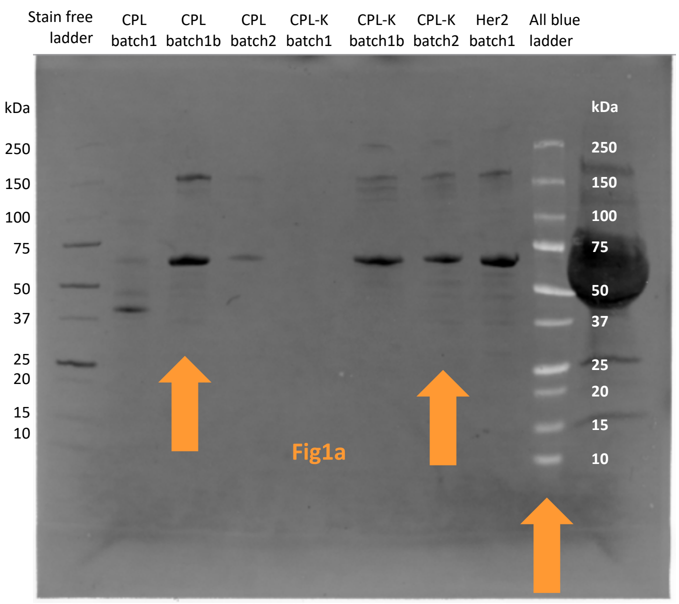

2016-12-08 19hr48min35sec

Source data for Fig.S1b:

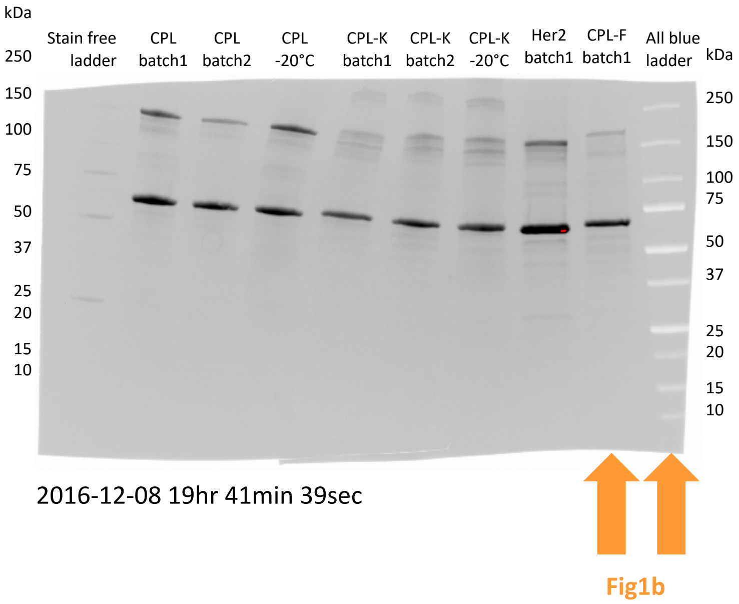

2016-12-08 19hr 41min 39sec

B. Figure 6

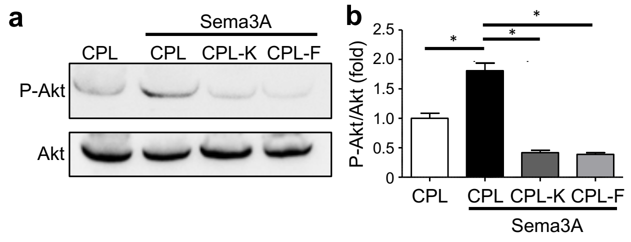

Figure 6a shows the results of staining the same protein extracts with two different antibodies, one specific for Akt and one specific for P-Akt. The Westernblot xperiment was performed using the same nitrocellulose membrane, that was cut before incubation with the antibodies. Figure 6b shows the densitometric quantification of the bands seen with Act and P-Act antibody. The results are displayed as fold change in the relative amount of P-Akt versus Akt (ratio of the densitometric quantification of the bands seen with P-AKt antibody to Akt; the densitometric data were normalized to the amount of protein in the lanes of the blot) which was normalized to the relative amount of P-Akt over Akt in the control (CPL), which was set to 1. The statistics for quantification provided in the figure is based on densitometric analysis of Western blots from three independent experiments.

The Westernblot experiment shown in in Figure 6a was performed by loading two halves of a Biorad stain-free SDS-PAGE gel with the same samples. Following electrophoresis, the gel was blotted to membrane, imaged under UV light, and cut into the two halves:

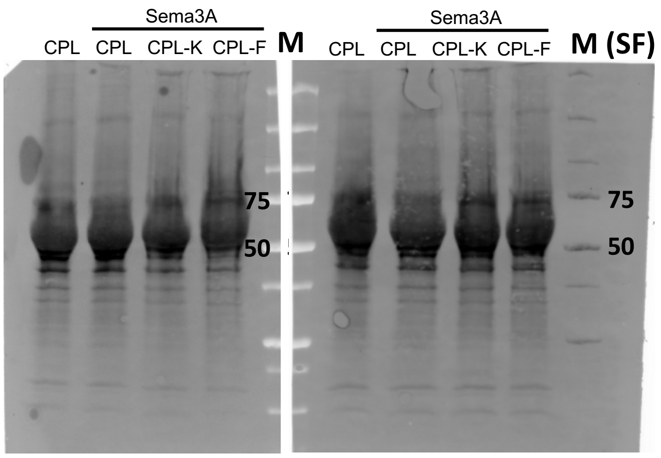

The left half and the right half of the filter were then incubated separately with antibodies for the specific detection of Akt and P-Akt, respectively, and developed for luminescent detection:

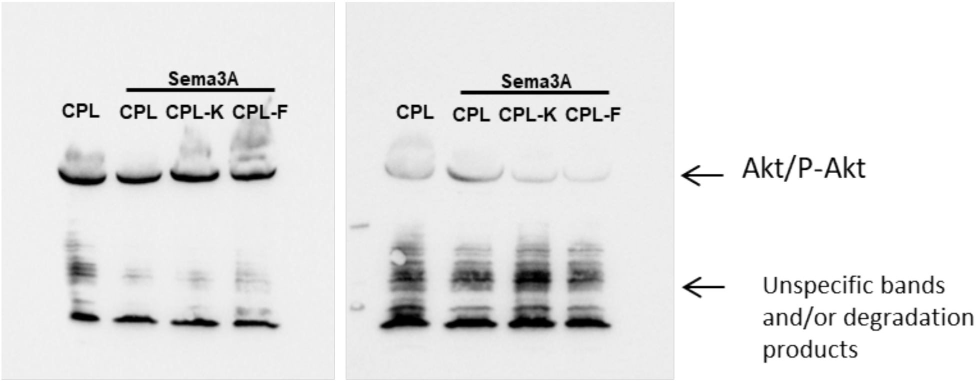

Anti-Akt Anti-P-Akt

To verify the molecular size of the detected Akt and P-Akt bands, the stain-free and immunoblot images were merged: to co-visualize the Akt / P-Akt bands with the size marker (next page):

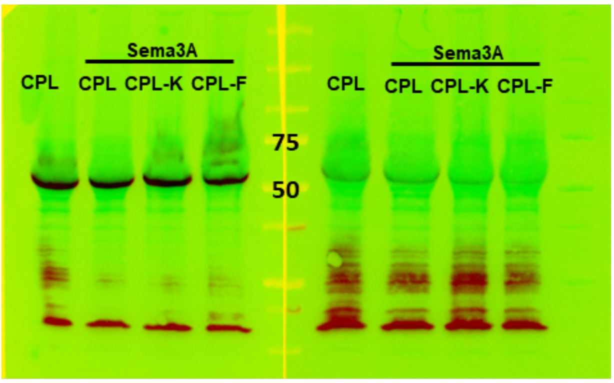

Merge

The two additional replicate experiments (Experiment 2 and Experiment 3) were combined. Here samples from the second experiment and the third experiment were each combined in two stain free SDS-PAGE gels of which one was incubated with anti Akt antibody and second was incubated with anti-P-Akt antibody.

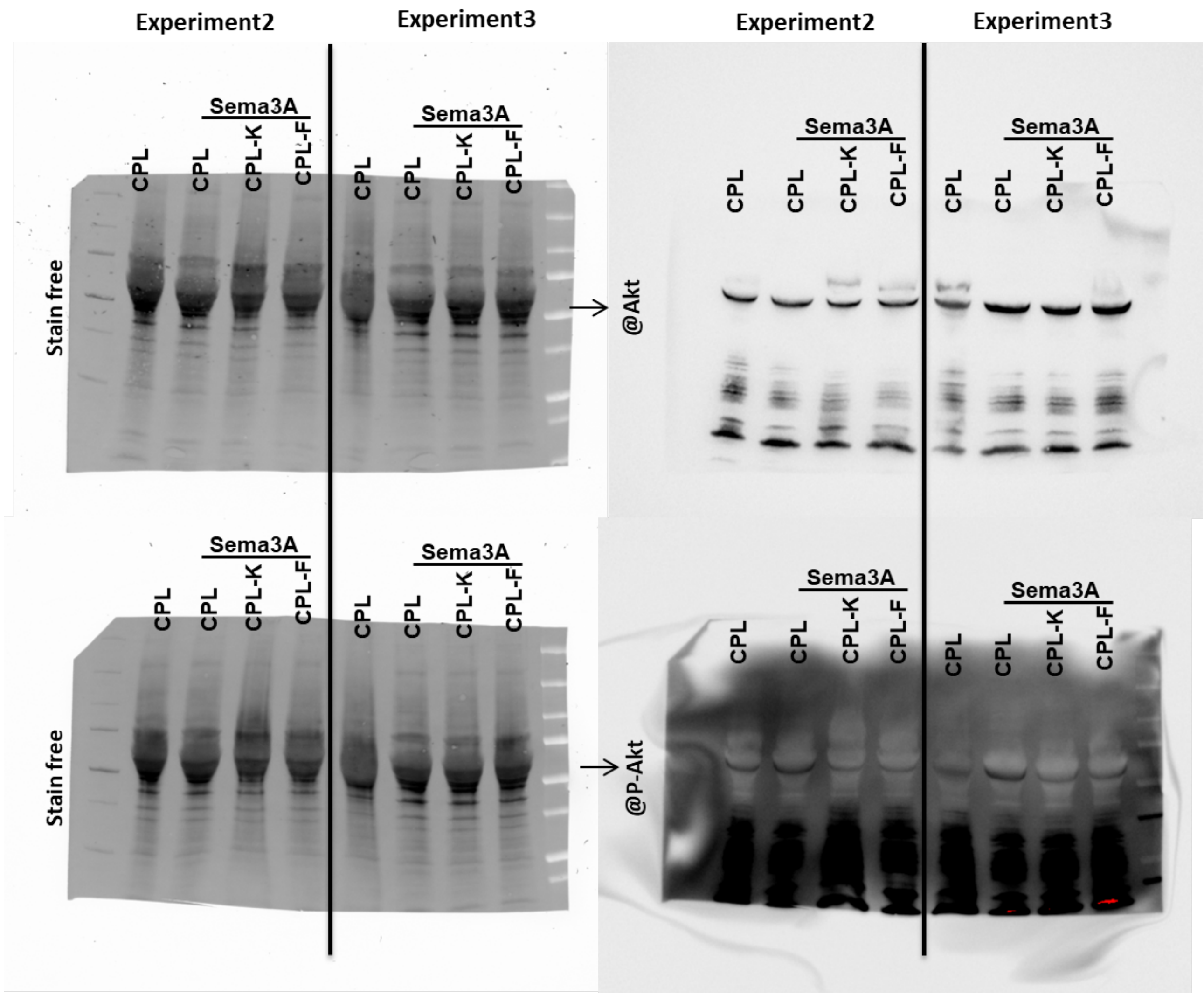

The bands in Westernblots were quantified with Biorad Image Lab Software:

	lane	Akt			P-Akt			P-Akt/Akt
		Volume (Int)	Norm. Factor	Norm. Vol. (Int)	Volume (Int)	Norm. Factor	Norm. Vol. (Int)	
Experment 1	CPL1	52252817	1	52252817	399035750	1	399035750	7,636636126
	CPL sema1	41670402	0,941425	39229571	478485000	1,163228	556587149,6	14,18794892
	CPLK sema1	51773168	0,980361	50756398	178266100	0,972284	173325276,8	3,414845884
	CPLF sema1	39527470	0,924094	36527081	82760650	1,161332	96112591,19	2,631269419
Experment 2	CPL2	18144404	1	18144404	109402436	1	109402436	6,02954145
	CPL sema2	22270119	0,789664	17585911,25	200682150	0,986867	198046591,3	11,26166216
	CPLK sema2	23340644	0,985822	23009720,35	82707012	0,887471	73400074,65	3,18995944
	CPLF sema2	25537307	0,99191	25330710,19	81373046	1,008687	82079933,65	3,240332902
Experment 3	CPL3	18495542	1,101011	20363795,19	166300959	0,987791	164270590,6	8,066796441
	CPL sema3	18216225	0,740838	13495271,7	190734760	0,982507	187398236,8	13,88621445
	CPLK sema3	45759160	0,963985	44111143,85	107049187	1,017992	108975216	2,470469057
	CPLF sema3	58820372	0,969202	57008822,18	120027756	1,211812	145451075,1	2,551378357

P-Akt/Akt	CPL	CPL + Sema3A	CPLK + Sema3A	CPLF + Sema3A
Experment 1	7,636636126	14,18794892	3,414845884	2,631268291
Experment 2	6,02954145	11,26166216	3,18995944	3,240332902
Experment 3	8,066796441	13,88621445	2,470469057	2,551378357
mean	7,244324672			

P-Akt/Akt normalized to mean of CPL	CPL	CPL + Sema3A	CPLK + Sema3A	CPLF + Sema3A
Experment 1	1,054154317	1,958491587	0,471382225	0,363217886
Experment 2	0,832312427	1,554549619	0,440339105	0,447292612
Experment 3	1,113533256	1,916840434	0,341021306	0,352189952
mean	1	1,809960547	0,417580878	0,387566816
